# Therapeutic Advancements Across Clinical Stages in Melanoma, With a Focus on Targeted Immunotherapy

**DOI:** 10.3389/fonc.2021.670726

**Published:** 2021-06-10

**Authors:** Claudia Trojaniello, Jason J. Luke, Paolo A. Ascierto

**Affiliations:** ^1^ Unit of Melanoma, Cancer Immunotherapy and Development Therapeutics, Istituto Nazionale Tumori IRCCS Fondazione G. Pascale, Napoli, Italy; ^2^ Cancer Immunotherapeutics Center, University of Pittsburgh Medical Center and Hillman Cancer Center, Pittsburgh, PA, United States

**Keywords:** immunotherapy, advanced melanoma, immune system, target therapy, immune checkpoint inhibitor (ICI)

## Abstract

Melanoma is the most fatal skin cancer. In the early stages, it can be safely treated with surgery alone. However, since 2011, there has been an important revolution in the treatment of melanoma with new effective treatments. Targeted therapy and immunotherapy with checkpoint inhibitors have changed the history of this disease. To date, more than half of advanced melanoma patients are alive at 5 years; despite this breakthrough, approximately half of the patients still do not respond to treatment. For these reasons, new therapeutic strategies are required to expand the number of patients who can benefit from immunotherapy or combination with targeted therapy. Current research aims at preventing primary and acquired resistance, which are both responsible for treatment failure in about 50% of patients. This could increase the effectiveness of available drugs and allow for the evaluation of new combinations and new targets. The main pathways and molecules under study are the IDO inhibitor, TLR9 agonist, STING, LAG-3, TIM-3, HDAC inhibitors, pegylated IL-2 (NKTR-214), GITR, and adenosine pathway inhibitors, among others (there are currently about 3000 trials that are evaluating immunotherapeutic combinations in different tumors). Other promising strategies are cancer vaccines and oncolytic viruses. Another approach is to isolate and remove immune cells (DCs, T cells, and NK cells) from the patient’s blood or tumor infiltrates, add specific gene fragments, expand them in culture with growth factors, and re-inoculate into the same patient. TILs, TCR gene transfer, and CAR-T therapy follow this approach. In this article, we give an overview over the current status of melanoma therapies, the clinical rationale for choosing treatments, and the new immunotherapy approaches.

## Introduction

In recent years, we have witnessed a revolution in treatment and, consequently, a marked improvement in the overall survival (OS) of patients with metastatic melanoma. Before 2011, treatment with chemotherapy had been the standard of care for melanoma patients; the median survival of patients who were diagnosed with advanced melanoma was 6–9 months, with only 25% alive at 1 year and <10% alive at 5 years (1). Since 2011, with the approval of several agents for the treatment of advanced melanoma, the likelihood of survival for patients with advanced disease has increased. The therapeutic armamentarium in melanoma now comprises immune checkpoint inhibitors (ICIs) and targeted therapy in the adjuvant and metastatic settings, and these agents are also being investigated in the pre-surgical setting. Tumor cells are able to evade immune surveillance in some ways, including the activation of immune checkpoint pathways that suppress the antitumor immune response, and overexpress the ligand for PD-1 (programmed cell ligand PD-1 or PD-L1), which facilitates the escape from the immune system (2).

Antibodies, such as nivolumab or pembrolizumab (anti-PD-1), can reinstitute an intra-tumoral immune response by targeting PD-1, interrupting the co-inhibitors’ signature pathways and inducing the immune response against cancer cells (3). The MAPK signaling pathway also plays a pivotal role in the advancement of melanoma (4, 5). Its activation triggers a signal cascade, which leads to the inactivation of MAPK, including RAS (HRAS, NRAS, and KRAS), RAF serine/threonine kinases (ARAF, BRAF, and CRAF), MEK, and ERK. Important cellular activities such as differentiation, proliferation, survival, migration, and angiogenesis are regulated by these kinases. If signaling through this pathway is dysregulated, unconstrained cell growth and cell transformation can occur (6). The activation of *BRAF* mutations can be found in both skin (50%) and mucosal melanomas (10–20%) (7) and can cause constitutive activation of *BRAF* and downstream MAPK signaling (8). It has been demonstrated that patients affected by *BRAF*V600 mutation-positive unresectable or metastatic melanoma can be treated with the MEK inhibitors trametinib, cobimetinib, and binimetinib, and the BRAF inhibitors dabrafenib, vemurafenib, and encorafenib. *BRAF*-resistant melanomas usually determine a reactivation of the MAPK signaling pathway (9). This pathway can be used by the tumor as an “escape route” from the BRAF inhibitor; therefore, the addition of a MEK inhibitor allows to delay the development of resistance. Therapies that combine MEK and BRAF inhibitors have demonstrated to be more effective and to reduce the toxicity resulting from monotherapy with BRAF inhibitors. However, there is still large room for improvement in the treatment of metastatic melanoma by addressing two of its major problems: resistance and treatment-related adverse events (TrAEs). TrAEs, which are frequent in combination therapy, lead to treatment discontinuation in approximately 15% of patients and dose modifications or interruptions in approximately 50% of patients (10), while resistance is developed by 80% of patients within the first 3 years of therapy.

Some preclinical and clinical studies are ongoing, focusing on new combination treatments and new targets, with the aim to improve the outcome of patients with melanoma. At present, there are approximately 2250 active trials testing more than 295 targets. Here, we comprehensively present current approaches for the treatment of metastatic melanoma in adjuvant, neoadjuvant, and metastatic settings.

## Adjuvant Treatment

### Stage III

#### Current State of Care

The treatment of choice for early-stage cutaneous melanoma is surgical excision, and, in most cases, it can be curative. However, some patients will ultimately relapse with metastatic or locally advanced disease. Clinical outcomes in patients with stage IIIB, C, and D have historically been poor, with a metastasis-specific survival (MSS) at 5 years of 83%, 69%, and 32%, respectively (11). Around 80% of relapses in resected stage III melanoma occurred within the first 2 years (12).

These differences have implications both in the clinical decision-making and in the design and analysis of clinical trials on adjuvant therapy.

At present, adjuvant treatment is indicated in patients at high risk of recurrence in patients with stage IIIB, C, and D or stage A with sentinel lymph node tumor deposits >1 mm.

Until 2012, IFN-α-2b was the only drug to demonstrate efficacy as adjuvant therapy in melanoma (11, 13). At present, IFN can only be considered in cases of stage IIB/C ulcerated melanoma, for which new-generation adjuvant therapies are not available, even if some clinical trials are ongoing.

Over the past years, some randomized studies in the adjuvant setting have been conducted to evaluate the activity of drugs that are already approved for metastatic disease (**Table 1**). Lymph node dissection has been included as the inclusion criteria in most adjuvant therapy trials; nevertheless, a lot of patients do not receive complete lymph node dissection anymore since the results of MSLT-2 (18). Moreover, the staging system for melanoma from the American Joint Committee on Cancer (AJCC) changed from the 7th to the 8th edition in January 2018 (12). The first adjuvant therapy study was the EORTC 18071, a randomized phase III trial that compared ipilimumab at a dosage of 10 mg/kg for 4 doses, then every 2 months for up to 3 years and placebo in patients with stage III (IIIA >1 mm lymph node metastasis, IIIB, IIIC) (19). This clinical trial showed a benefit of ipilimumab in terms of relapse-free survival (RFS), OS, and distant metastasis-free survival (DMFS) (14). However, this benefit was associated with a grade (G)3/4 adverse events (AE) rate of 54.1%, including a treatment-related mortality rate of 1% (n=5) for patients who received ipilimumab therapy (deaths from colitis, myocarditis, and Guillain-Barré syndrome) (19). Based on these data, ipilimumab was approved in 2015 by the US Food and Drug Administration (FDA) for the adjuvant treatment of melanoma, at a dosage of 10 mg/kg. In Europe, this treatment has never been approved. Currently, the use of ipilimumab in the adjuvant setting has been replaced by anti-PD-1 or BRAF-directed therapies.

**Table 1:** Update on the latest results of adjuvant clinical trials.

	EORTC 18071 (14)	COMBI – AD (15)	CheckMate 238 (16)	KEYNOTE 054 (17)
Stage	IIIA (>1 mm)/B/C	IIIA (>1 mm)/B/C	IIIB/C/resected IV	IIIA (>1 mm)/B/C
Treatment arm	Ipilimumab	Dabrafenib + trametinib	Nivolumab	Pembrolizumab
Control arm	Placebo	Placebo	Ipilimumab	Placebo
Update	7 years	5 years	4 years	3 years
RFS	39.2%	52%	51.7%	63.7%
OS	60%	65%	77.9%	NR
DMFS	44.5%	65%	59%	65%
trAEs G3–4	54.1%	31%	14.4%	7.7%

The COMBI-AD is a randomized, phase III trial comparing 12 months of adjuvant therapy with both the BRAF and the MEK inhibitors dabrafenib and trametinib, respectively, versus placebo in patients with resected, *BRAF*V600-mutant, stage III melanoma (14). At a minimum study follow-up of 60 months, the trial showed a benefit of targeted therapy in terms of RFS and DMFS (20). Based on these results, the combination was approved by the FDA in April 2018, followed by the EMA for the adjuvant treatment of patients with resected BRAFV600-mutant stage III melanoma.

A phase III clinical trial, the CheckMate 238, has compared nivolumab at a dosage of 3 mg/kg every 2 weeks for 1 year in patients with completely resected stage IIIB/C or IV with ipilimumab at a dosage of 10 mg/kg every 3 weeks (Q3W) for 4 doses and every 12 weeks thereafter for 1 year in the adjuvant setting (21). At a median follow-up of 51.1 months (16), the 4-year RFS was better in the nivolumab arm, while OS at 4 years was similar in both treatment groups (77.9 and 76.6%, respectively). Median OS (mOS) was not reached in both arms. However, 49% of ipilimumab-treated patients received subsequent therapy compared with 41% of those in the nivolumab group. TrAEs of G3/4 were reported in only 14.4% of patients in the nivolumab arm versus 45.9% of those in the ipilimumab arm, with discontinuation due to immune-related adverse events (IrAEs) in 9.7% and 42.6%, respectively (21). Two treatment-related deaths in the ipilimumab group were reported: marrow aplasia in one patient and colitis in one patient.

Based on this study, in December 2017, the FDA and then in July 2018, the EMA approved the use of nivolumab in the adjuvant melanoma setting in all stage III and IV resected patients. Similar RFS results were recently reported in phase III clinical trial KEYNOTE 054 (22) in which patients with stage III melanoma were randomized to treatment with pembrolizumab at a dosage of 200 mg Q3W or placebo. The 3-year RFS and 3–5-year DMFS were higher in the pembrolizumab group (17, 23). IrAEs of G3–4 occurred in 7.7% of patients in the pembrolizumab group and in 0.6% in the placebo group, and, in any case, the occurrence of an irAE was significantly associated with a longer RFS in the pembrolizumab arm (17). There was one pembrolizumab-related death (myositis) (22).

Based on this study, pembrolizumab was approved, first by the EMA in October 2018, then by the FDA in February 2019 for the adjuvant treatment of stage III melanoma patients.

#### Emerging Strategies

CA209-915 is a phase III, randomized clinical trial studying the effectiveness of adjuvant immunotherapy after complete resection of stage IIIB/C/D or IV melanoma, according to the AJCC 8th edition; drugs involved are nivolumab combined with ipilimumab versus ipilimumab or nivolumab monotherapy. Study enrollment has been completed, but full results have not yet been released. In November 2019, results for only one of the co-primary endpoints were announced. Data showed that the combination did not lead to any improvement in RFS in the all-comer (intent-to-treat) population (24). Previously, in November 2019, the supporting company reported that the combination did not result in a PFS improvement compared with nivolumab alone when used in the adjuvant setting for patients with resected stage IIB/C/D or stage IV melanoma and whose tumors expressed PD-L1 <1%, thus failing to achieve the co-primary endpoint of the trial (25).

IMMUNED is a randomized, double-blind phase II trial, evaluating adjuvant nivolumab plus ipilimumab versus nivolumab versus placebo in patients with resected stage IV melanoma. The primary endpoint was RFS. At a median follow-up of 28.4 months, the median RFS in the placebo group was 6.4 months and 12.4 months in the nivolumab group, whereas it was not reached in the combination group. In the nivolumab plus ipilimumab group, RFS at 2 years was 14% in the placebo group; versus 42% in the nivolumab group and 70% in the combination group. G3–4 TrAEs were reported in 27% of patients in the nivolumab group and in 71% of patients enrolled in the combination group (26).

In another phase IIb trial, patients with resected stage III/IV melanoma were randomized to receive tumor lysate, particle-loaded, dendritic cell (TLPLDC) vaccine versus placebo. By ITT analysis, 36-month OS was 76.2% versus 70.3% in placebo arm (HR: 0.72, p=0.437) and 36-month disease-free survival (DFS) was 35.6% *vs* 27.1% (HR: 0.95, p=0.84). By per-treatment analysis, 36-month DFS was 57.5% in TLPLDC arm versus 35% in the placebo group (HR: 0.50, p=0.025); this effect was more evident in resected stage IV patients, with a 36-month DFS of 60.9% versus 0% (HR: 0.12, p=0.001) (27). A phase III trial will evaluate the improvement of a TLPLDC vaccine as adjuvant treatment for resected stage IV melanoma, in combination with anti-PD-1 versus anti-PD-1 alone.

The SWOG 1404 is a phase III randomized study in stage IIIA (N2)/B/C or resectable IV melanoma in which patients will receive high-dose IFN or pembrolizumab (28). The primary endpoints are RFS and OS.

The CA045-022 is an ongoing phase III randomized, open-label trial, which compares patients with stage III or resected IV receiving adjuvant treatment with bempegaldesleuskin (NKTR-214), a PEGylated interleukin-2 (IL-2), in combination with nivolumab versus those on nivolumab alone (NCT04410445).

### Stage II

#### The Current State of Care

After excellent results were obtained with adjuvant treatment in patients with stage III melanoma, and the subsequent approval of nivolumab, pembrolizumab, and dabrafenib in combination with trametinib, attention has now shifted to stage II melanoma patients.

Patients affected by stage II melanoma are divided into two groups (low and high risk) according to the risk of relapse (**Table 2**) (29). Patients at low risk of recurrence (tumor ≤4 mm in thickness without ulceration or ≤2 mm in thickness with ulceration, stage IIA), have a high probability to be cured only by surgery. However, the 5-year MSS in stage IIC is 82%, which is comparable to the 83% of stage IIIB; patients with stage IIIA disease have a better prognosis than those with stage IIC disease.

**Table 2:** Low- and high-risk stage II melanoma (29).

	Low risk (stage IIA)	High risk (stage IIB/C)
Thickness	≤2 mm + ulceration ≤4 mm without ulceration	>2 mm + ulceration >4 mm (regardless ulceration)
Lymph node involvement	No	No
Melanoma-specific survival at 5 years (19)	94%	85% \ 82%
Melanoma-specific survival at 10 years (19)	88%	82% \ 75%

In countries without access to clinical trials, adjuvant (PEG)-IFN-α-2b treatment is an option for patients with ulcerated melanomas without palpable nodes (stage IIB/C) or stage III (30).

#### Emerging Strategies

The KEYNOTE-716 is one of the largest clinical trials currently ongoing. It is a phase III, randomized trial evaluating 1 year of pembrolizumab Q3W versus placebo in patients with stage IIB/C melanoma according to the AJCC 8th edition. The primary endpoint is RFS, and crossover from placebo or re-challenge of pembrolizumab is allowed (NCT03553836).

CA209-76K is a phase III, randomized, double-blind study of nivolumab versus placebo for 12 months after complete resection of stage IIB/C melanoma. In the event of disease recurrence, participants will have the option to receive on-study open-label nivolumab treatment (NCT04099251).

So far, the only trial, in development, evaluating adjuvant sequential treatment with BRAF (encorafenib) and MEK (binimetinib) inhibitors followed by anti-PD-1 versus anti-PD-1 alone versus placebo in melanoma stage IIA/B/C patients is the EORTC 1902.

## Neoadjuvant Therapy

### Current State of Care

At present, no neoadjuvant treatment is approved for patients with melanoma.

The ESMO Consensus Conference positively evaluated the data on neoadjuvant therapy for resectable stage III melanoma, although they did not justify the indication at the moment. However, if any agents would become available and associated with improved survival, it should be considered prior to surgical resection. When the disease is technically resectable but in-transit and/or bulky nodal, and surgery could be associated with major morbidity, neoadjuvant strategies should be considered even outside the context of a clinical trial (31).

Emerging strategies Preclinical studies suggested that neoadjuvant ICI treatment, compared with adjuvant treatment, is associated with antigen-specific T-cell responses. The primary site of the tumor can be used as a spring of antigens for the spread and activation of tumor-specific T cells and to control micro-metastases (32); however, optimal regimens have not been defined (33).

#### Intravenous Treatment

A study presented at the 2019 ASCO Annual Meeting and conducted in institutions participating in the International Neoadjuvant Melanoma Consortium pooled data from six neoadjuvant systemic therapy trials (anti-PD-1 in 133 patients and BRAF/MEK target therapy in 55 patients). It demonstrated how patients treated with neoadjuvant systemic therapy (nivolumab, either as monotherapy or in addition with ipilimumab, pembrolizumab, or dabrefenib plus trametinib) had better chances to be relapse-free when achieving pathologic complete response (pCR) compared with those who did not achieve it. pCR was achieved in 41% of patients (38% treated with immunotherapy and 47% with targeted therapy). Immunotherapy was more effective than targeted therapy at 12 months; 83% of the patients who received it remained relapse-free compared with just 65% of those who underwent targeted therapy. Patients with pCR showed an improved RFS compared to those without pCR. Moreover, 100% of the patients with pCR who were treated with immunotherapy were relapse-free versus just 72% of those without pCR (p<0.001). Targeted therapy at 12 months showed a relapse-free rate of 88% in pCR patients and 43% in patients without pCR (34).

Some clinical trials evaluated the role of neoadjuvant treatment in patients with melanoma:

The OpACIN trial was the first to evaluate neoadjuvant treatment in patients with melanoma (35). This was a randomized phase 1b trial, in high-risk stage III melanoma patients, which compared neoadjuvant nivolumab plus ipilimumab followed by nivolumab and ipilimumab after regional lymph node dissection versus adjuvant ipilimumab plus nivolumab. In both arms, 90% of patients experienced G3/4 trAEs.

At the 4-year follow-up, all of the AEs have recovered to grade ≤1 except endocrine toxicities requiring hormone replacement therapy, and no new G3–4 AEs were observed (36).

Pathologic response (pR) was achieved in 78% of patients in the neoadjuvant arm, with three pCRs, three near pCRs (≤10% viable tumor cells), and one patient achieving a pathological PR (partial response) (pPR ≤50% viable tumor cells). After a mean follow-up of 36 months for OpACIN, only one out of 71 patients (1.4%) relapsed on neoadjuvant therapy with pathological response (pR) (37). After a median follow up of 48 months, none of the seven patients with a confirmed pPR in the neoadjuvant arm have relapsed. The estimated 4-year RFS rate for the neoadjuvant arm was 60% and 60% for the adjuvant arm, and the 4-year OS was 90% and 70%, respectively (36). The OpACIN trial is the first to show how neoadjuvant combination is superior to adjuvant immunotherapy. Furthermore, this trial suggested that pR can function as a surrogate marker for RFS.

Another phase II trial (38) of neoadjuvant treatment enrolled 23 patients with high-risk stage III or oligometastatic stage IV melanoma in two arms: neoadjuvant with four courses of nivolumab versus three courses of ipilimumab in combination with nivolumab, followed by surgical resection and subsequently by adjuvant nivolumab for 6 months. Combination treatment showed high response rates (overall response rate [ORR]: 73%, pCR 45%) but a high rate of grade 3 trAEs (73%). Treatment with nivolumab monotherapy showed moderate responses (ORR 25%, pCR 25%) with a low incidence of grade 3 toxicity (8%), without grade 4 or 5 trAEs. At a median follow-up of 15.6 months, 11/11 of the patients receiving dual checkpoint blockade were still alive. Due to disease progression in 17% of patients in the monotherapy arm and a high rate of grade 3 trAES, the study was stopped early. The combination of ipilimumab with nivolumab resulted in a trend to improved survival outcomes (PFS, DMFS, OS) compared with nivolumab monotherapy, although significance was not reached (38).

OpACIN-neo is a phase II, open-label, randomized trial in high-risk stage III melanoma. In this trial, 86 patients were randomized to one of three neoadjuvant dosing schedules (arm A: 2 × ipilimumab 3 mg/kg + nivolumab 1 mg/kg Q3W; arm B: 2 × ipilimumab 1 mg/kg + nivolumab 3 mg/kg Q3W; and arm C: 2 × ipilimumab 3 mg/kg Q3W followed immediately by 2 × nivolumab 3 mg/kg Q2W for 6 weeks prior to surgery, without adjuvant therapy (39). The primary endpoints were both the proportion of patients with grade 3/4 IrAEs within the first 12 weeks and the rate of patients achieving a radiological objective response and pR at 6 weeks. Arm C was closed early due to high-grade G3/4 AEs. At 24-month follow-up, of the 81 patients alive, 68% still showed irAEs but only 3% experienced ≥grade 3 irAEs (40). Radiologic objective response and pR were reported in 63% and 80% in group A, in 57% and 77% in group B and in 35% and 65% in group C, respectively. OpACIN-neo identified that the treatment regimen in group B, two cycles of ipilimumab 1 mg/kg plus nivolumab 3 mg/kg once Q3W intravenously, can be considered as the most suitable dosing and schedule, associated with the lowest grade G3/4 toxicities and a similar pR rate compared with the other two dosing regimens.

Estimated 24-month RFS was 84% for all patients (95% CI: 76–92%); 90% for arm A (95% CI: 80–100%), 78% for arm B (95% CI: 63–96%) and 83% for arm C (95% CI: 70–100%), thus confirming the high pR rates achieved with combination in neoadjuvant setting (41).

“Nadina study” is a yet-to-start, randomized, international phase III trial, which will evaluate two courses of neo-adjuvant ‘low-dose’ ipilimumab (1 mg/kg) + nivolumab (3 mg/kg) followed by surgery and then 1 year of anti-PD-1 adjuvant systemic therapy. The PRADO study was an extension cohort of the OpACIN-neo study, which aims to further evaluate the pR rate and toxicity of combination treatment of nivolumab and ipilimumab in the neoadjuvant setting for two cycles and to save patients from surgery on the basis of pR (42). In this study, all patients did receive excision of the index node. Patients that achieved major pR (MpR) in the largest lymph node metastasis, did not undergo lymph node dissection while patients with pPR or with no pathologic response underwent lymph node dissection followed by nivolumab or target adjuvant therapy of 52 weeks. pCR was achieved in 50% of patients, near pCR in 11%, and pPR in 10%. The ORR was 71%. This meant that a complete therapeutic lymph node dissection was needed by just 40 out of 99 patients, thus reducing surgical morbidity. Longer follow-up is necessary to fully evaluate safety and RFS in patients without lymph node dissection (43).

At the ESMO 2020 were reported health-related quality of life data showing that patients with MpR following neoadjuvant immunotherapy who have reduced the extent of surgery have a significantly better health-related quality of life scores (44).

#### Oral Treatment

Dabrafenib and trametinib were evaluated in the neoadjuvant setting in a single-center, open-label, randomized, phase II trial on 21 patients with surgically resectable clinical stage III or oligometastatic IV melanoma with *BRAF*V600E/K mutations (45). Patients were randomized to receive the neoadjuvant/adjuvant treatment or the standard surgery ± adjuvant therapy. Patients assigned to the targeted therapy arm received 8 weeks of neoadjuvant dabrafenib and trametinib followed by surgery and then adjuvant dabrafenib and trametinib for up to 44 weeks.

An interim safety analysis showed how treatment with neoadjuvant dabrafenib plus adjuvant trametinib allowed longer event-free survival compared with the standard approach; the trial was thus stopped early. The study is now continuing as a single-arm study of neoadjuvant plus adjuvant dabrafenib and trametinib. Event-free survival, the primary endpoint of the trial, was 19.7 months for neoadjuvant plus adjuvant dabrafenib and trametinib, versus 2.9 months for standard care (HR: 0.016, 95% CI: 0.00012–0.14; p<0.0001), without any G4 AEs in either arm.

NeoCombi was a single-arm, open-label, single-center, phase II trial, which enrolled patients who were affected by stage IIIB/C, *BRAF* V600-mutated melanoma and receiving dabrafenib plus trametinib for 12 weeks of neoadjuvant therapy before surgery, followed by 40 weeks of adjuvant therapy (46). The primary endpoints were the rate of patients achieving a pCR and the proportion of patients achieving a response at week 12. At a median follow-up of 27 months, 86% achieved a RECIST (Response Evaluation Criteria in Solid Tumors) response (46% CR and 40% PR), 14% achieved a stable disease without progression in any patients. After surgery, all patients achieved a pR (49% pCR and 51% non-complete pR). A 2-year RFS in patients with a complete pR was achieved in 63.3% versus 24.4% of patients with a non-complete pR. Serious trAEs occurred in 17% of patients and 29% of patients developed G3–4 AEs (most common were pyrexia and syncope), without treatment-related deaths.

#### Intratumoral Treatment

The oncolytic virus Talimogen laherparepvec (T-VEC) consists of a genetically modified herpes simplex virus (HSV-1) able to preferentially thrive in neoplastic cells: it enhances antigen loading of MHC class I molecules and promotes the expression of granulocyte–macrophage colony-stimulating factor (GM-CSF), increasing tumor antigen presentation by dendritic cells.

The administration of T-VEC prior to surgery was associated with improved RFS and OS compared with surgery alone in patients with resectable advanced melanoma, according to results of a multicenter, open-label, phase II trial (47). In this study, patients with high-risk stage IIIB-IV M1a resectable melanoma were randomly assigned to immediate surgery or intralesional T-VEC followed by surgery. Among the patients in the T-VEC arm, 22.8% had a pCR. Investigator-assessed clinical response in the T-VEC arm was 13.2%. The disease control rate (DCR) was 40.8%. The most common trAEs were flu-like symptoms; G3 AEs in the T-VEC group consisted of two cases of cellulitis and one case each of anembryonic gestation, cholecystitis, device occlusion, influenza, and wound infection [49]. The 3-year OS rate in T-VEC arm was 83.2% versus 71.6% for surgery alone (HR: 0.54, 80% CI: 0.36–0.83; p=0.061). The 3-year RFS rate was 46.5% with T-VEC plus surgery compared with 31% with surgery alone (HR: 0.67; 80% CI: 0.51–0.88, p=0.043). Median OS at 3 years was not reached in both arms (48).

The Neo-C-Nivo is a phase II study that evaluates the effects of neoadjuvant intra-tumoral CMP-001 in combination with nivolumab in patients with stage IIIB/C/D treatment-naïve melanoma, deemed surgically resectable, with an accessible tumor for biopsy and CMP-001 injection (49). CMP-001 is a type A CpG packaged with a virus-like particle that activates tumor-associated plasmacytoid dendritic cells *via* TLR9 inducing type I IFN and anti-tumor CD8+ T cells (50). The primary endpoint was the MpR and incidence of dose-limiting toxicities, while RFS, OS, and radiographic response were the secondary endpoints. At the final analysis presented at SITC 2020, no dose-limiting toxicities or G4/5 trAEs were observed; the most frequent G3 AE was hypertension (9.7%) followed by arthralgia in 3.2%, colitis in 3.2%, hypophosphatemia in 3.2%, and injection site infection in 3.2% of patients. Radiographic responses were seen in 43%, while 30% had stable disease (SD) and 27% had progressive disease (PD). pCR was achieved in 50% and pMR in 10% with a pR of 70%. Responders had evidence of activated CD8+ T cells peripherally and TIM-3 upregulation was evidenced on CD8+ T cells in non-responders. The RFS at 1 year was 90% in all pathological responders, with a median RFS not reached in pathological responders versus 5 months in non-pathological responders (51).

## Treatment of Stage III/IV Non-Resectable Melanoma

### Current State of Care

The treatment of patients with metastatic or unresectable melanoma has greatly evolved in the last decade, thanks to the development of ICIs and MAPK molecular targeted therapy directed towards the oncogenic BRAF and MEK signaling pathways (**Tables 3** and **4**).

**Table 3:** Last results of KEYNOTE 006, CheckMate 066 and CheckMate 067 trial.

	KEYNOTE 006 (53)	CheckMate 066 (54)	CheckMate 067 (55)
**Treatment arm**	**Pembrolizumab**	**Nivolumab**	**Nivolumab + ipilimumab**
Last update	5 years	5 years	5 years
mPFS	8.4	5.1	11.5
PFS	NR	28%	36%
mOS	32.7	37.3	NR (more than 60)
OS	38.7%	39%	52%
ORR	42%	42%	58%
trAEs G3–4	17%	15%	59%

**Table 4:** Last results of Combi-D, Combi-V, CoBRIM, Columbus and Imspire 150 trial.

	Combi-D (62)	Combi-V (62)	coBRIM (66)	Columbus (67)	Imspire 150 (68)
Treatment arm	Dabrafenib + trametinib	Dabrafenib + trametinib	Vemurafenib + Cobimetinib	Encorafenib + binimetinib	Vemurafenib + Cobimetinib + Atezolizumab
Control arm	Dabrafenib	Vemurafenib	Vemurafenib	Encorafenib Vemurafenib	Vemurafenib + cobimetinib
Last update	5 years	5 years	5 years	4 years	2 years
mPFS	11.1	11.1	12.6	14.9	15.1
PFS	17%	20%	14%	26%	43.5%
mOS	25.9	25.9	22.5	33.6	28.8
OS	32%	36%	31%	39%	76.7% (2 years)
ORR	68%	68%	68%	64%	66.3%
trAEs G3–4	54%	62%	60%	68%	79%

#### Immunotherapy

The first immunotherapeutic agent approved in metastatic melanoma was ipilimumab, in 2011 (52), based on a randomized phase III (52) clinical trial “MDX010-020” in which patients with metastatic melanoma, pretreated, were randomly assigned to receive ipilimumab, gp100 (peptide vaccine), or ipilimumab in combination with gp100. OS was significantly longer with ipilimumab alone or in combination with gp100 (10.1 months) compared with gp100 alone (6.4 months; HR: 0.68, p<0.001).

In 2014, the results of the randomized phase III trial KEYNOTE 006 (56), which evaluated patients with advanced melanoma receiving pembrolizumab (10 mg/kg Q2W Q3W) or ipilimumab (3 mg/kg Q3W for 4 total doses), accounted for the FDA approval of pembrolizumab (53).

In 2015, nivolumab was approved in untreated patients with metastatic melanoma, based on the results of CheckMate 066 (57), a randomized double-blinded phase III study that evaluated treatment with nivolumab versus dacarbazine in patients with advanced *BRAF* wild-type melanoma in the first-line setting (54).

In 2016, the combination of nivolumab plus ipilimumab in untreated patients with *BRAF* V600 wild-type and *BRAF* V600 mutation-positive metastatic melanoma was approved following the results of the randomized double-blind phase III study CheckMate 067 (55), which compared the combination regimen versus nivolumab or ipilimumab in monotherapy in the first-line treatment of patients with advanced melanoma.

In patients with unresectable cutaneous, subcutaneous, or nodal melanoma, T-VEC was approved in 2015 by the FDA, based on the OPTiM phase III randomized trial (58). In this study, patients with stage IIIB or IV melanoma were randomly assigned to intralesional T-VEC or GM-CSF administered subcutaneously at 125 µg/mq daily for 14 days in 28-day cycles. At a median follow-up of 49 months, mOS was 23.3 months with TVEC and 18.9 months with GM-CSF (p=0.051), in the ITT population estimated OS probability at 5 years was 33% versus not evaluable, durable response rate (DRR) was 19.0 versus 1.4%; ORR was 31.5 versus 6.4%, respectively. In T-VEC patients, the median time to CR was 8.6 months; median CR duration was not reached (59). The subanalysis of the OPTiM trial comparing stage IV M1b or M1c patients with metastases to visceral or lung sites and patients with melanoma at stage IIIB/IV M1a, revealed as DRR of 5% versus 25%, ORR of 9% versus 41%, and CR rates of 4% versus 17%, respectively (60). Therefore, it seems that only stage IIIB–IV M1a disease may be addressed with TVEC monotherapy, likely due to its activity against dermal satellite or in transit metastases and its high degree of control locoregional disease; in stage M1b or M1c systemic effects, which normally require combination approaches, are limited.

#### Targeted Therapy

At present, there are three combination regimens approved.

The combination of dabrafenib plus trametinib was the first BRAF–MEK combination approved for metastatic melanoma, in 2015, based on the two phase III clinical trials, COMBI-v (61, 62) and COMBI-d (63), comparing the combination with vemurafenib or dabrafenib monotherapies, respectively. The randomized, phase III co-BRIM trial compared the combination of vemurafenib and cobimetinib versus vemurafenib monotherapy (64). In 2015, the FDA approved this combination to treat patients with metastatic melanoma (64, 65). COLUMBUS was a phase III, randomized trial addressing the combination of encorafenib and binimetinib versus vemurafenib (66, 67), thus gaining the approval from the FDA in 2018.

In terms of efficacy, the approved BRAFi/MEKi combinations are overall comparable; response rates range from 60 to 70% and 18-month PFS rates range from 30 to 40% (63, 64, 69) with distinct toxicity profiles.

On the basis of the findings from IMspire150, in 2020, the FDA approved the PD-L1 inhibitor atezolizumab in combination with cobimetinib and vemurafenib for the treatment of patients with advanced melanoma with *BRAF* V600 mutations. The trial was a double-blind, multicenter, placebo-controlled randomized phase III study that enrolled patients with unresectable locally advanced melanoma or previously untreated *BRAF* V600 mutation-positive metastatic melanoma (68).

### Emerging Strategies

#### Alternative Dosing of Ipilimumab + Anti-PD-1

A remarkably interesting phase II study evaluated the need for more than two doses of nivolumab plus ipilimumab followed by maintenance nivolumab, in patients with unresectable stage III/IV melanoma, with the aim to evaluate whether decreasing the dose of the combination would reduce toxicity achieving the same outcome than that of the standard approach (70). Patients were treated with two doses of nivolumab (1 mg/kg) plus ipilimumab (3 mg/kg) followed by a CT scan at week 6; if tumor burden growth was >4%, patients received two further doses of nivolumab plus ipilimumab; if otherwise, patients underwent maintenance nivolumab therapy. Results showed that 68% of patients had tumor shrinkage or no growth at week 6 with two doses of the combination. None of the patients who had PD (32% of patients) at week 6 moved on the response at week 12, showing that efficacy and toxicities with the combination appear to be driven by the first two doses of treatment; however, it is still unclear which patients are at increased likelihood of benefitting from fewer doses. Furthermore, no difference in toxicity was disclosed, with emerging toxicity deemed as probably related to early combination dosages (70).

The CheckMate 511 trial, evaluating two different dosages (ipilimumab 3 mg/kg and nivolumab 1 mg/kg or ipilimumab 1 mg/kg and nivolumab 3 mg/kg) of the nivolumab plus ipilimumab combination, showed similar results. The primary aim of the study was to determine if ipilimumab 1 mg/kg was better tolerated than the 3 mg/kg dosage. Remarkably, the lower dose of ipilimumab combined with 3 mg/kg of nivolumab was associated with a more favorable tolerability profile than the higher dose (G3/5 toxicity of 34% in the ipilimumab 1 mg/kg arm *vs* 48% in the ipilimumab 3 mg/kg arm; p=0.006). The two regimens did not appear to differ in secondary efficacy endpoints. The ipilimumab 3 mg/kg arm had a slightly numerically higher objective response rate than the ipilimumab 1 mg/kg arm (50.6% *vs* 45.6%), but median PFS and 12-month OS were perfectly comparable in the two arms (71).

In a retrospective study, Da Silva et al. showed that patients resistant to PD-1 monotherapy treated with combination (ipilimumab plus anti-PD-1) had a RR of 31% versus ipilimumab alone 12% (p<0.01), with a PFS and OS at 1 year 27% and 57%, respectively, versus 13% and 38% (p<0.01) (72). OS at 18 months was 53% versus 25%, and mOS was 20.4 versus 8.8 months, respectively. In *BRAF* wild-type patients, RR was higher with combinations versus ipilimumab alone (38% *vs* 9%, p<0.01), while RR was similar, 19% versus 24% in *BRAF*-mutated patients, respectively. AEs ≥3 was similar with combination therapy (30%) or monotherapy (34%, p=0.48) and were not associated with response (72).

In a phase II trial, combination therapy with low-dose ipilimumab with pembrolizumab demonstrates marked antitumor activity in patients with melanoma following PD on a PD-1 antibody. Patients received pembrolizumab 200 mg intravenously Q3W plus ipilimumab 1 mg/kg Q3W for 4 doses. Pembrolizumab was continued in monotherapy for up to 2 years. The primary endpoint, RR, was 31%. Median PFS was 5.0 months (95% CI: 2.8–8.3) and median OS was 24.7 months (95% CI: 15.2–undetermined). Grade 3–4 trAEs were reported in 15 (27%) of 70 patients enrolled in the study, the most common being diarrhea, rash, and transaminase elevation (73).

#### Loco-Regional Treatment

Talimoneg laherparepvec (T-VEC), an oncolytic virus, was evaluated in patients with advanced melanoma in a phase II study in combination with ipilimumab versus ipilimumab alone. A total of 38 patients (39%) in the combination arm and 18 patients (18%) in the ipilimumab arm had an odds ratio (OR: 2.9; 95% CI: 1.5–5.5; p=0.002). Responses comprise both injected and visceral lesions; specifically, the latter decreased in 52% of patients in the combination arm and 23% of patients in the ipilimumab arm. More common AEs included fatigue, chills, and diarrhea. The incidence of G ≥3 AEs was 45% and 35%, respectively (74). At the interim analysis at 4 years (n=198), median follow-up was 48.3 months for combination and 35.7 months for ipilimumab alone. DRR improved for combination versus monotherapy (33.7% *vs* 13.0%; OR: 3.4; 95% C: 1.7–7.0; p=0.001). Median PFS was 13.5 months with combination and 6.4 months with ipilimumab alone (HR: 0.81; 95% Cl: 0.57–1.15; p=0.23). Median OS was not reached for combination and was 50.1 months for ipilimumab (HR: 0.82; 95% CI: 0.54–1.25; p=0.36). In a subgroup analysis, patients without the *BRAF* V600 mutation receiving the combination therapy showed improved DRR and PFS (DRR: 33.9% *vs* 5.0%; median PFS: 18.0 *vs* 4.5 months); DRR in *BRAF* V600 mutation-positive patients were similar between arms (34.3% *vs* 26.5%; mPFS: 4.2 months *vs* 6.4 months). No additional safety signals were observed in follow-up (75).

In the Masterkey 265 phase Ib trial, T-VEC was evaluated in combination with pembrolizumab in patients with unresectable stage IIIB/IVM1c melanoma (76). The CR rate was 43%. In total, 12/13 responders (92.3%) were still in response, including all nine patients with a CR. Kaplan–Meier estimates of 4-year PFS and OS rates were 55.9% and 71.4%, respectively. Patients who achieved a CR or PR had better OS (p=0.0056) compared with those who did not respond. Median OS was not reached for responders and was 24.4 months for non-responders (77).

Tilsotolimod (IMO 2125), a synthetic Toll-like receptor 9 agonist (TLR9) oligonucleotide, changes the tumor microenvironment by acting on dendritic cells and macrophages and is being evaluated in multiple solid tumors. Type 1 IFN responses induced by local drugs determine in both injected and non-injected lesions an increased downstream T-cell activation and proliferation and antigen presentation (78). ILLUMINATE-204 is a phase I/II trial in patients with advanced melanoma refractory to anti-PD-1 therapy of intratumoral tilsotolimod in combination with ipilimumab (79). In 49 patients with anti-PD-1 refractory melanoma and evaluable for efficacy, investigators reported ORR of 22% with 71% DCR, mOS was 21 months, the median duration of response (mDOR) was 11.4 months. Tumor reduction was observed in both injected and noninjected tumors. trAEs of G3/4 were reported in 48% of patients, the most common serious trAEs were autoimmune hepatitis, hyponatremia, and hypophysitis (80).

In a phase III trial, ILLUMINATE 301, tilsotolimod at a dosage of 8 mg in combination with ipilimumab was well tolerated and showed durable and substantial clinical benefit (NCT02644967).

SD-101, a TLR-9 agonist, was assessed in a phase Ib/II study, at multiple doses injected in a single tumor in combination with pembrolizumab in patients with advanced melanoma naïve to anti-PD-1 treatment (81). This combination showed promising response rates compared with those expected with pembrolizumab alone. Frequently observed G ≥3 trAEs were myalgia 9%, headache 9%, fatigue 9%, chills 7%, and malaise 5% (81). ORR in the 2 mg group was 71% (95% CI: 57–82; CR: 13%) and in the 8 mg group was 49% (95% CI: 33–65; CR: 7%) with responses in both injected and non-injected lesions, including visceral. PFS was higher in the 2 mg group with median PFS in 2 mg not reached, in the 8 mg arm PFS was 10.4 months. The 6-month PFS and OS rates were 81% and 98% in the 2 mg arm and 60% and 92% in the 8 mg arm, respectively (81).

Findings from a phase Ib trial studying the intratumoral TLR9 antagonist CMP-001 were presented at SITC 2019. This agent pushes tumor-associated plasmacytoid dendritic cells to produce interferon and has been shown to produce durable responses when administrated in combination with pembrolizumab for patients with PD-1-resistant metastatic melanoma (82). The best ORR in patients treated with pembrolizumab and CMP-001 was 23.5%, while CMP-001 alone resulted in a lower ORR of 11.5%. Intratumoral CMP-001 was well-tolerated and provided both local and distant responses in patients with advanced melanoma reporting disease progression on prior PD-1 blockade. CMP-001 monotherapy induced systemic tumor regression in some patients, but the duration of response was substantially increased by the addition of pembrolizumab (83).

Electroporated plasmid IL-12 (TAVO or tavokinogene telseplasmid) is a novel immuno-modulating intratumoral therapy, which delivers IL-12 into the tumor microenvironment; it has been shown to synergize with anti-PD-1 antibodies in patients progressed to anti-PD-1. Although the IL-12/IFN-γ axis is usually not active in advanced melanoma, intratumoral electroporation of pIL-12 can recover this axis, favoring anti-PD-1 immunotherapy activity in patients unresponsive to anti-PD-1 treatment (84, 85).

KEYNOTE 695 is a phase II trial evaluating the combination of plasmid IL-12 (TAVO) with pembrolizumab in patients with advanced melanoma refractory to PD-1 treatment. Results showed that the combination is associated with an ORR of 30% (95% CI: 18.0–43.6%), with 6% of patients achieving a CR. In patients with M1c/M1d disease, the ORR was slightly higher (35.3%). Patients who had previously received treatment with ipilimumab showed the highest ORR with the treatment (40%). The median DOR was 12.2 months (95% CI: 5.6–not evaluable). The most common trAEs were fatigue (26.8%), procedural pain (23.2%), diarrhea (19.6%), nausea (10.7%), and rash (10.7%). Three patients had grade 3 toxicities: cellulitis, enteritis, and Lichen planus (86).

PVSRIPO is a novel immunotherapy consisting of a non-neurovirulent poliovirus chimera that activates innate immunity that is injected directly into tumors. The recombinant oncolytic poliovirus is designed to infect antigen-presenting cells, such as macrophages and dendritic cells that express CD155. The poliovirus receptor is often expressed on malignant cells of solid neoplasia, as well as in myeloid and endothelial cells. This strategy was evaluated in a phase I trial in patients with unresectable melanoma progressed after PD-1 and BRAF/MEK therapy (if *BRAF* mutated). Two of four (50%) patients with in-transit disease had pCR. At a median follow-up of 12 months, 50% (6/12) patients remained progression free. All AEs were of G1/2 severity, with pruritis (50.0%) and erythema (33.3%) being the most common trAEs. Around 33% of patients achieved a response (87).

#### Triplet Therapy

Combinations of immunotherapy and targeted therapy in the treatment of metastatic melanoma suggest even greater benefits in survival than the two approaches alone. Data show that patients who receive the triplet of anti PD-L1 plus MEK inhibitor and BRAF inhibitor have a greater number of T cells in the tumor environment (BRAF inhibitors can increase the ability of T cells, triggered by immunotherapy, to penetrate the tumor) (88). Indeed, targeted therapies could help prevent the spread of cancer, while immunotherapy stimulates the immune system to attack cancer cells.

The phase I/II KEYNOTE-022 (89) trial addressed the safety and efficacy of dabrafenib and trametinib in combination with pembrolizumab in untreated patients with unresectable or metastatic *BRAF*-mutated melanoma. It could not meet its primary endpoint of improved median PFS, although a trend to a longer PFS with pembrolizumab (median PFS 16.0 months compared to 10.3 months with placebo) was reported. An updated analysis of KEYNOTE 022 at 24-month follow-up showed the triplet potential as a treatment option for patients with *BRAF*-mutant advanced melanoma (90). 24-month PFS rates were 41% with pembrolizumab versus 16.3% with placebo; mOS was not reached with pembrolizumab versus 26.3 months with placebo (HR: 0.64; 95% CI: 0.38–1.06). OS rates at 24 months were 63.3% *versus* 51.7%, respectively (HR: 0.64). mDOR was 25.1 months in the pembrolizumab group *versus* 12.1 months in the placebo group. G3–5 AEs occurred in 58.3% of the pembrolizumab group *versus* 25% of the placebo group. The most common G3–5 trAEs were pyrexia (10.0% *versus* 3.3%), increased aspartate aminotransferase (6.7% versus 3.3%), and increased γ-glutamyl transferase (6.7% *versus* 5.0%) (90).

TRIdent is a phase II trial of nivolumab in combination with dabrafenib and trametinib in patients with metastatic melanoma with *BRAF* mutations, refractory to ICI therapy, and in patients with asymptomatic brain metastasis (91). Of the 27 patients, after a median follow-up of 18 months, ORR was 92% (3 CR, 12%), median PFS was 8.5 months, and mDOR was 5.8 months. Around 78% of patients experienced G3–4 trAEs (92).

IMspire170 is a phase III, multicenter, open-label, randomized study, which evaluated cobimetinib plus atezolizumab compared with pembrolizumab in treatment-naive patients with advanced *BRAF*V600 wild-type melanoma (NCT03273153). In the primary analysis, with a median follow-up of 7 months, the combination treatment did not significantly improve the primary endpoint of median PFS compared with pembrolizumab (5.5 versus 5.7 months). ORR was 26% with cobimetinib plus atezolizumab versus 32% with pembrolizumab; DCR was 46% versus 44%. mOS was not reached in either arm (HR: 1.06; 95% CI: 0.69–1.61). Grade ≥3 AEs occurred in 67% versus 33% of patients; AEs lead to discontinuation of all treatments in 12% versus 6% (93).

COMBI-I is a randomized, double-blind, placebo-controlled, phase III study comparing the combination of anti-PD-1 spartalizumab (PDR001) in combination with dabrafenib and trametinib versus the combination of placebo with dabrafenib and trametinib, in previously untreated patients with unresectable or metastatic *BRAF*V600 mutation-positive melanoma (94). This trial failed to meet the primary endpoint of investigator-assessed PFS according to an update on the phase III COMBI-i trial (95).

#### Emerging Pathways

##### Anti-LAG-3

Another immune checkpoint is the lymphocyte activation gene 3 (*LAG3*). It is a marker of T-cell exhaustion and negatively regulates their functions.

Initial efficacy of anti-LAG-3 antibody in combination with nivolumab in melanoma patients who progressed on or after prior treatment with anti-PD-1/PD-L1 was reported in a phase I/II trial, showing ORR of 16% and DCR of 45% (96). CA224-047 is a randomized phase II/III trial studying previously untreated metastatic or unresectable melanoma the effects of relatlimab (anti-LAG-3) in combination with nivolumab versus nivolumab alone (NCT03470922). ORR is the primary endpoint for the phase II component, while PFS is the primary endpoint for phase III. Other endpoints include OS, DOR, DCR, safety, and tolerability. The results are not yet available (97).

A first-in-human phase I dose-finding study is evaluating the antibody MK-4280 directed against LAG-3 both in combination with pembrolizumab and as monotherapy for advanced solid tumors (98).

IMP321 (eftilagimod alpha) may lead to stronger antitumor CD8 T-cell responses compared to pembrolizumab monotherapy thanks to the activation of the dendritic cell network and the subsequent T-cell recruitment at the tumor site. This is due to the fact that IMP321 is a LAG-3Ig fusion protein, and, as an MHC class-II agonist, it activates antigen-presenting cell and CD8 T cells. The TACTI-mel study is evaluating the use of eftilagimod alpha. In this multicenter, open-label, dose-escalation, phase I study there was an increase in activated CD8 and CD4 T-cell counts. ORR was observed in 33% of patients refractory PD-1 and in 50% of anti-PD-1-naive patients. The main AE was for reactions at the injection site and there were no reports of dose-limiting toxicities (99).

##### HDAC-Inhibitors

The immune system can be modulated by an increase of antigen expression on neoplastic cells and suppression of regulatory cells such as myeloid-derived suppressor cells and regulatory T cells. HDAC inhibitors can act on these two mechanisms and thus contrast resistance to checkpoint inhibition (100).

Entinostat (ENT) is an oral class I-selective histone deacetylase inhibitor. In the tumor microenvironment, it leads to the downregulation of immunosuppressive cell types showing synergy with anti-PD-1 inhibition in preclinical models (101). Encore-601 is an open-label phase Ib/II study evaluating ENT in combination with pembrolizumab in patients with recurrent or metastatic melanoma who progressed on or after anti-PD-1 therapy. Of the 53 evaluable patients, nine had a PR and one had a CR, with an ORR of 19% (95% CI: 9–32%). Median PFS was 4.2 months. At data cutoff, the mDOR was 12.5 months. The most common G3–4 AEs included neutropenia, fatigue, and hyponatremia (102).

Tucidinostat (HBI-8000) was evaluated in a phase Ib/II study in combination with nivolumab in patients with unresectable or advanced melanoma who were anti PD-1-naïve. The most common trAEs included fatigue, diarrhea, abdominal pain, and lymphopenia. The most frequent G ≥3 AEs were hypophosphatemia, neutropenia, thrombocytopenia and lymphopenia. ORR was 74% among 31 patients (four CR and 19 PR), with five SD and three PD. The median time to response was 1.9 months (103).

##### TIGIT

T-cell suppression, production, and activation, as well as tumor cell immune evasion and the inhibition of antiviral immune responses, can be regulated by immunomodulatory receptors, such as TIGIT (104). The anti-TIGIT antibody MK-7684 was studied in a multicenter phase I trial in combination with pembrolizumab. It was used in 34 patients with advanced solid tumors for whom standard treatment options had failed. The ORR was 19%, and the DCR was 47%. AEs occurred in 53% of monotherapy and 65% of combination therapy (105).

##### Multikinase Inhibitor

Lenvatinib (LEN) is a multikinase inhibitor of VEGFR 1−3, FGFR 1−4, PDGFRα, RET, and KIT. In preclinical studies, LEN increased infiltration of CD8+ T-cell, decreased tumor-associated macrophage populations, and improved inhibitor activity of PD-1 (106). In a multicenter phase Ib/II trial, LEN was evaluated in combination with pembrolizumab. ORR was 47.6% (95% CI: 25.7–70.2). mDOR was 12.5 months, mPFS was 7.6 months, PFS rate at 12 months was 38.3%. G3 trAEs occurred in 62% of patients, with no fatal trAEs. Most common any-grade trAEs were fatigue (52%), decreased appetite (48%), diarrhea (48%), hypertension (48%), dysphonia (43%), and nausea (43%) (107).

LEAP-004 is a phase III, single-arm, study in which patients with unresectable stage III/IV melanoma who progressed on anti-PD-1 treatments, received pembrolizumab plus lenvatinib. At a median follow-up of 12 months, the ORR was 21.4%, and 43.7% of patients achieved SD. In patients previously treated with anti-CTLA-4 plus anti-PD-1/PD-L1, the ORR was 31%. The mDOR was 6.3 months, with 72.6% of patients still responding at 6 months. mPFS was 4.2 months, and at 9 months the PFS rate was 26.2%. mOS was 13.9 months, with 65.4% of patients alive at 9 months. G ≥3 was reported in 44.7%, moving to treatment discontinuation in 7.8%. The most common AEs were hypertension (56.3%), diarrhea (35.9%), nausea (34.0%), and hypothyroidism (33.0%) (108).

##### IDO

Indoleamine 2,3 dioxygenase-1 (IDO) is an enzyme that when overexpressed in the tumor microenvironment makes it immunosuppressive. There are some preclinical data showing that inhibition of IDO leads to a more immunogenic tumor microenvironment (109, 110).

Inhibition of the IDO-1 enzyme can be achieved with a specific and potent oral inhibitor, such as epacadostat (111), which is being evaluated in combination with ant-PD-1 in multiple tumors type. The open-label phase I/II trial (ECHO-202/KEYNOTE-037) studying epacadostat plus pembrolizumab in patients with advanced melanoma showed promising anti-tumor activity. A total of 64 patients were enrolled in this trial. The ORR was 56% (CR: 14%), and the DCR was 71%. mPFS was 12.4 months, and 18-month PFS was 49%. Among treatment-naïve patients with advanced disease treated with 100 mg of epacadostat, ORR was 58% (CR 8%), and the DCR was 74%. Epacadostat plus pembrolizumab showed a favorable safety profile, with a 20% incidence of related G3/4 toxicity (112). Similarly, in the open-label phase I/II ECHO-204 study of patients with advanced solid tumors, epacadostat plus nivolumab was well tolerated and showed promising activity. In the 30 patients with advanced melanoma not previously treated, eight patients were treated with epacadostat 100 mg and 22 with 300 mg. In the first group, ORR was 75% and DCR was 100%. Preliminary DCR in the group treated with epacadostat 300 mg was 64% (113). After a median follow-up of 417 days, ORR was 62% and DCR was 78%. In treatment-naive patients, ORR was 65% and DCR was 80%; PFS at 6 and 12 months was 77% and 63%, respectively (median not reached); and the OS at 12 months was 92% (median not reached). More frequently reported grade ≥3 trAEs were present in 48% in patients treated with high dose (300 mg) versus 13% in patients treated with low dose (100mg), the most common G ≥3 were rash and ALT increase. Eight patients in the arm treated with 300 mg discontinued treatment due to trAEs. There were no AE-related deaths (114).

The phase III ECHO/KEYNOTE-252 evaluated epacadostat plus pembrolizumab versus permbolizumab alone. However, no improvement in PFS and OS in unresectable stage III/IV melanoma was reported and the study was stopped after the second interim analysis with a median follow-up of 12.4 months. mPFS was 4.7 months in the combination group versus 4.9 months in the pembrolizumab group (HR: 1.00, p=0.52). mOS was not reached in either group. No differences in outcome were observed regarding to PD-L1, IDO1, or *BRAF* mutation status (115).

##### Pegylated IL-2

A CD122-preferential IL-2 pathway agonist, such as mempegaldesleukin (BemPEG; NKTR-214), is able to increase T-cell clonality, PD-1 expression, and tumor-infiltrating lymphocytes (116). Furthermore, adding BemPEG to nivolumab can convert baseline tumors from PD-L1 negative to PD-L1 positive (117).

In the PIVOT-02 phase I/II trial, patients with previously untreated metastatic melanoma were treated with the combination of BemPEG plus nivolumab (118). At a median follow-up of 18.6 months, the ORR was 53% (CR 34%). At a median follow-up of 29 months, mPFS for the entire cohort of 30.9 months (95% CI: 5.3–not estimable), and OS was not reached (119). Responses were not dependent upon PD-L1 expression at baseline. On-treatment biomarkers (CD8+ and eosinophils) predicted response to the combination, well before radiographic evidence. The most common G1/2 trAEs were flu-like symptoms (80.5%), rash (70.7%), fatigue (65.9%), pruritus (48.8%), nausea (46.3%), arthralgia (43.9%), decreased appetite (36.6%), and myalgia (36.6%). Registrational phase III trials evaluating BemPEG plus nivolumab are enrolling subjects in first-line metastatic melanoma treatment (CA045-001; NCT03635983).

##### New Engineered CTLA-4 Antibodies

MGD019 is a bispecific, Fc-bearing (IgG4) DART (dual-affinity re-targeting antibody) molecule that blocks PD-1 and CTLA-4 and shows higher activity on dual PD-1/CTLA-4-expressing cells. T-cell responses *in vitro* can be improved by MGD019 to levels reached by a combination of nivolumab and ipilimumab. This drug was evaluated in a phase I trial in patients with advanced solid tumors, demonstrating an acceptable safety profile and encouraging early evidence of anti-tumor activity. trAEs occurred in 78.8% of patients, most commonly fatigue, nausea, arthralgia, pruritus, and rash (120).

Ipilimumab non-fucosylate (NF) and ipilimumab–probody are two engineered CTLA-4 derivatives being evaluated plus nivolumab versus nivolumab alone in phase I/II trial in advanced solid cancers (NCT03994601 and NCT03369223).

##### STING Pathway

The Sting pathway has been recognized as a major stimulator of dendritic cells (121). A phase I dose-escalation and dose-expansion clinical trial in patients with advanced, metastatic treatment-refractory solid tumors was designed to evaluate the safety, tolerability, and clinical activity of the novel stimulator of IFN genes (STING) pathway ADU-S100. The most common trAEs were pyrexia, injection site pain, diarrhea, and headache. The combination of ADU-S100 and spartalizumab demonstrated antitumor activity in anti-PD-1-naïve triple-negative breast cancer and in melanoma formerly treated with immunotherapy; among the 25 melanoma patients radiologically evaluable for efficacy, two previously immunotherapy-treated melanoma patients achieved PR (NCT0317293). Following administration, a rise in systemic cytokines including MCP-1, IFN-β, and IL-6 were observed. This indicates target engagement of ADU-S100 and activation of the STING pathway. In a subset of patients, a rise in CD8+ T cells in injected tumors was observed in on-treatment tumor biopsies (122).

##### T-Cell Therapy

T-cell therapies, such as tumor-infiltrating lymphocyte (TIL) therapy, T-cell receptor (TCR) therapy, and CAR T-cell therapy, which have shown preliminary signals of activity, are ready to have an important impact in metastatic melanoma.

###### TIL

The TIL therapy being studied in an investigational immunotherapy study is named lifileucel (LN-144). The adoptive cell transfer therapy used in this study involves patients receiving a lymphocyte depleting preconditioning regimen, prior to infusion of autologous TIL, followed by the administration of IL-2. C-144-01 is a multicenter phase II study that evaluated the efficacy and safety of lifileucel in patients with metastatic melanoma who received at least one prior systemic therapy including an ICI and a BRAF inhibitor (if *BRAF* mutated). There were four cohorts in the study in which patients received different forms TIL therapy. In cohort 4, which received second-generation cryopreserved TILs, at a median follow-up of 5.3 months, the ORR observed was 32.4%. The DCR was 72.1% (123). Findings from cohort 4 were consistent with those reported in cohort 2, which received cryopreserved TILs. The investigator-assessed ORR for cohort 2 was 36.4% after a median follow-up of 18.7 months, the DCR was 80.3%, and responses occurred regardless of the location of the resected tumor (124). The most common any-grade AEs observed were thrombocytopenia (89.4%), chills (80.3%), and anemia (68.2%). The most common G3/4 AEs were thrombocytopenia (81.8%), anemia (56.1%) and febrile neutropenia (54.5%). Lifileucel is likely to be approved by the FDA and will likely become part of standard practice in the future.

###### TCR

A different approach in cancer treatment is the use of therapies exploiting genetically modified T-cells, such as T-cell receptor–engineered T-cell therapy and chimeric antigen receptor T-cell therapy, melanoma antigen recognized by T cells (MART-1), tyrosinase, and glycoprotein (gp100), as well as most antigens, are primarily found in normal melanocytes and melanomas (125, 126).

The melanoma antigene gp100 can be targeted with the first-in-class bispecific fusion protein tebentafusp; this happens through a high-affinity T-cell receptor (TCR) binding domain and an anti-CD3 T-cell engaging domain, redirecting T cells to kill tumor cells that express gp100. It is being evaluated in a phase I/II trial in metastatic melanoma. Tebentafusp was generally well-tolerated and active in both patients with metastatic uveal melanoma and patients with metastatic cutaneous melanoma. Patients in both cohorts achieved a 1-year OS rate of 65%. Cytokine measurements during treatment were consistent with the induction of markers related with IFN-γ pathway in the tumor and periphery (127). A high-affinity MART-1-specific TCR for TCR gene therapy in metastatic melanoma was evaluated in a phase I trial in patients with metastatic melanoma (128).

A phase II trial of lymphodepleting chemotherapy followed by autologous TILs ± dendritic cells vaccine and high-dose IL-2 for patients with metastatic melanoma is ongoing (129). Patients were randomized to receive TIL alone or TIL plus dendritic cells pulsed with MART-1 peptide. In this trial, also patients with brain metastasis were included (56%). Treatments were well tolerated with no G5 AEs. There were no toxicities conferred by the dendritic cell vaccination. The ORR was 63% (5/8) in TIL + dendritic cell arm (one CR, four PR) and 40% (4/10) in TIL arm alone (one CR, three PR; P=0.64). There was no difference in survival between the arms. The median PFS was 3.6 months in the TIL arm and 7.2 months in the TIL+ dendritic cell arm, while the median OS was 4.1 years in the TIL arm and 2 years in the TIL + dendritic cell arm (130).

## Conclusion

Metastatic melanoma has long been recognized as an immunologically affected tumor refractory to cytotoxic chemotherapy. With better characterization and understanding of the complex pathophysiology of the melanoma, the past decade has seen the progress of multiple therapies: checkpoint inhibitors and targeted therapy radically changed the prognosis of melanoma. These agents transformed the treatment of metastatic melanoma, demonstrating efficacy in a significant percentage of patients. However, what we have learned over the years is that not all patients can achieve the same benefit; in fact, if 50% of patients can be considered to be cured, there are always 50% who continue to not respond to the treatments available or develop resistance leading to tumor progression. Novel strategies are needed to further advance the standard of care, the immediate challenge is therefore to try to understand the resistance mechanisms, both primary and secondary, which prevents about 50% of patients from benefiting from these treatments, in order to increase the effectiveness of the drugs available by evaluating innovative approaches with the combination of different molecules. Novel targeted agents, particularly targeted immunotherapy, have shown great promise. The impressive results seen for targeted immunotherapy, as well as their potential use in combination regimens due to a tolerable side-effect profile, suggest many promising new paths for advancing the standard of care in refractory melanoma. The main pathways and molecules under study are investigated in this review: IDO inhibitor, TLR9 agonist, STING, oncolytic viruses, LAG-3, HDAC inhibitors, pegylated IL-2 (there are currently about 3000 trials that are evaluating immunotherapeutic combinations in different tumors). An additional emerging and very promising immune treatment is the adoptive T-cell therapy, which consists of TILs, engineered TCR therapy, and CAR-T. The goal of these treatments is to improve the cytotoxicity of cytotoxic T cells, to enhance tumor regression. TILs therapy requires the isolation of tumor-infiltrating lymphocytes from the tumors, expansion by IL-2 treatment, and reinfused into the patients with additional IL-2 treatment. TIL therapy in metastatic melanoma patients showed ORR ≥50%, with 22% of complete remission (131, 132). In the next few years, the possibility of treating melanoma patients at an early stage (i.e., in an adjuvant and neoadjuvant context) with the presence of new combinations in patients who are refractory to first-line therapies in the metastatic setting, may increase the percentage of patients who can be cured.

## Author Contributions

CT, JL, and PA wrote and approved the manuscript. All authors contributed to the article and approved the submitted version.

## Funding

JL acknowledges the Department of Defense Career Development Award (W81XWH-17-1-0265), the Sy Holzer Endowed Immunotherapy Research Award, and the Hillman Fellowship for Innovative Cancer Research.

## Conflict of Interest

PA has/had a consultant/advisory role for Bristol Myers Squibb, Roche-Genentech, Merck Sharp & Dohme, Novartis, Array, Merck Serono, Pierre-Fabre, Incyte, Medimmune, AstraZeneca, Syndax, Sun Pharma, Sanofi, Idera, Ultimovacs, Sandoz, Immunocore, 4SC, Alkermes, Italfarmaco, Nektar, Boehringer-Ingelheim, Eisai, Regeneron, Daiichi Sankyo, Pfizer, Oncosec, Nouscom, Takis, Lunaphore, Seagen. He also received research funding from Bristol Myers Squibb, Roche-Genentech, Array, Sanofi, and travel support from MSD. JL declares Scientific Advisory Board: (no stock) 7 Hills, Spring bank (stock) Actym, Alphamab Oncology, Arch Oncology, Kanaph, Mavu, Onc.AI, Pyxis, Tempest. Consultancy with compensation: Abbvie, Array, Bayer, Bristol-Myers Squibb, Checkmate, Cstone, Eisai, EMD Serono, KSQ, Janssen, Merck, Mersana, Nektar, Novartis, Pfizer, Regeneron, Ribon, Rubius, Silicon, Tesaro, TRex, Werewolf, Xilio, Xencor. Research Support: (all to institutions for clinical trials unless noted) AbbVie, Agios (IIT), Array (IIT), Astellas, Bristol-Myers Squibb (IIT & industry), Corvus, EMD Serono, Immatics, Incyte, Kadmon, Macrogenics, Merck, Moderna, Nektar, Numab, Replimmune, Rubius, Spring bank, Synlogic, Takeda, Trishula, Tizona, Xencor. Travel: Pyxis. Patents: (both provisional) Serial #15/612,657 (Cancer Immunotherapy), PCT/US18/36052 (Microbiome Biomarkers for Anti-PD-1/PD-L1 Responsiveness: Diagnostic, Prognostic, and Therapeutic Uses Thereof).

The remaining author declares that the research was conducted in the absence of any commercial or financial relationships that could be construed as a potential conflict of interest.
